# Intracranial Subdural Hygroma: A Rare Complication of Failed Neuraxial Anesthesia?

**DOI:** 10.7759/cureus.37090

**Published:** 2023-04-04

**Authors:** Duarte Filipe Lopes Simões Duarte, Catarina Costa, Diana Gomes, Nelson Gomes, Marcos Pacheco da Fonte

**Affiliations:** 1 Anesthesiology, Centro Hospitalar de Entre Douro e Vouga, Santa Maria da Feira, PRT

**Keywords:** subdural hygroma, neuraxial anesthesia, post dural puncture headache, obstetric anesthesia, dural puncture, neuraxial anesthesia complications, hygroma

## Abstract

The unintentional puncture of the dura during epidural procedures is a noteworthy and prevalent complication in obstetric patients. Early recognition can be difficult, particularly when attempts at neuraxial anesthesia are unsuccessful. Rare intracranial complications, such as subdural hematomas and subdural hygromas, can occur after dural puncture and they should be suspected in the presence of atypical headaches or other neurological symptoms. We describe a case of a woman who had an unrecognized dural puncture following failed neuraxial anesthesia and later presented with symptoms of intracranial hypotension. Urgent investigation with cranial CT scan revealed two intracranial subdural hygromas. We discuss the diagnosis, follow-up, and successful management of this case with an epidural blood patch. It is crucial to maintain a high level of suspicion for complications after neuraxial anesthesia and to have a low threshold for imaging and investigation to prevent unfavorable or fatal consequences.

## Introduction

Epidural anesthesia in obstetric patients can frequently be complicated by dural puncture [1]. Dural punctures can sometimes go unnoticed during the procedure and, if not recognized early, may lead to various neurological disorders that are associated with significant morbidity [1-3]. Severe headache, typically orthostatic, is the most common symptom. The prevailing hypothesis is that it results from intracranial hypotension, arising from the leakage of cerebrospinal fluid (CSF) through a persistent breach in the dura mater [2-4].

Intracranial lesions, such as subdural hematomas and subdural hygromas, although rare, are also possible complications of dural puncture [2,4-8]. The underlying mechanism is thought to be brain displacement secondary to CSF leakage, with consequent stretching and tearing of the intracranial subdural veins [5,8]. Such lesions may present with variable neurological signs and symptoms, which make the diagnosis and management challenging, especially in the absence of a history of dural puncture [2].

We describe a case of intracranial subdural hygromas secondary to unrecognized dural puncture following multiple unsuccessful attempts at neuraxial anesthesia for labor, its follow-up, and benign resolution after treatment with an epidural blood patch.

## Case presentation

A 37-year-old, American Society of Anesthesiologists (ASA) grade II, female patient with medicated hypothyroidism and a history of depression and migraines, underwent general anesthesia for an elective cesarean section, following three unsuccessful attempts at neuraxial anesthesia. A few hours after the surgery, the patient developed a persistent holocranial orthostatic headache, which was particularly severe in the temporal regions, and was accompanied by photophobia. Despite these symptoms, the patient was discharged on the third postoperative day, with nonsteroidal anti-inflammatory drugs (NSAIDs) and an acetaminophen prescription.

**Figure 1:**
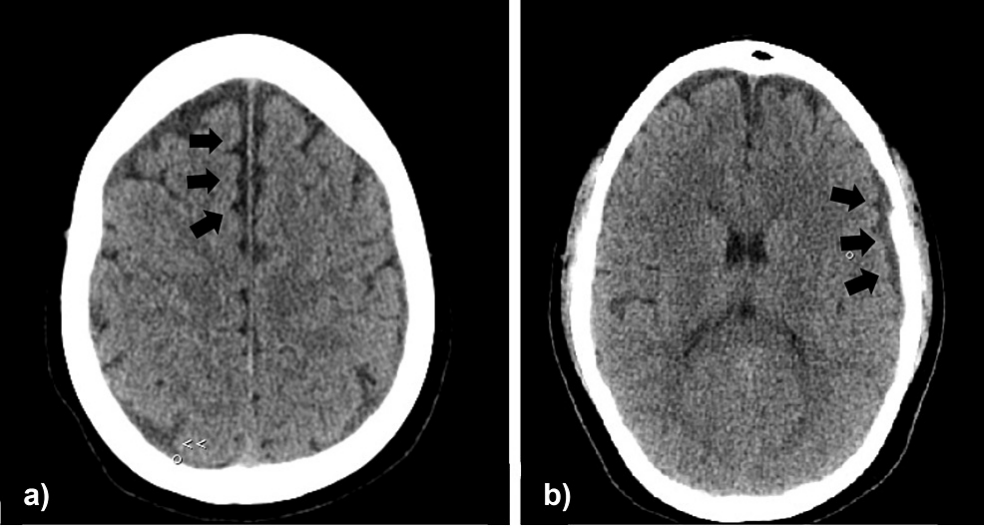
Cranial CT showing (a) parasagittal parietal and (b) frontoparietal hygromas (black arrows).

On the sixth postoperative day, along with the persistent headache, the patient developed diplopia and strabismus, which prompted her to seek medical attention at the emergency department the following morning (seventh postoperative day). During the physical examination, convergent strabismus with paresis of the sixth cranial nerve, as well as paresthesia of the right hemiface was detected, without facial paresis and other signs or symptoms. An urgent cranial computed tomography (CT) scan was conducted, which revealed the presence of a left frontoparietal hygroma measuring 4.6 mm, as well as a right upper parasagittal parietal hygroma measuring 6 mm (Figure 1). The images showed no significant mass effect and normal brain parenchyma. An additional CT angiography of the cerebral arteries revealed no abnormalities. For treatment, an epidural blood patch was recommended, and after the elucidation of its benefits and risks, the patient refused. Some pain relief was achieved with intravenous analgesics and fluid administration, but the strabismus persisted. Despite being informed of her condition and the potential risks, the patient left the hospital against medical advice.

**Figure 2:**
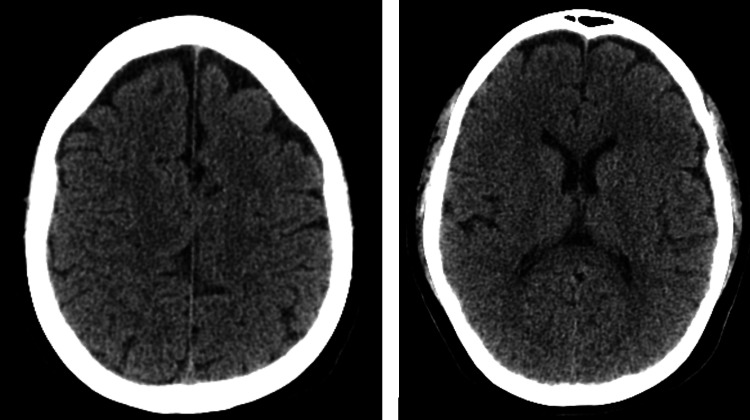
Follow-up cranial CT showing partial resolution of the parasagittal parietal and frontoparietal hygromas.

On the following day, the patient returned, as her symptoms did not improve, and she was admitted for treatment. She agreed to undergo an epidural blood patch, which was successfully performed at the same level as the initial neuraxial technique attempts (L3-L4), using 18 mL of autologous blood, with no reported complications. After 12 hours of bed rest, there was some headache improvement, but bilateral sixth cranial nerve paresis persisted. After repeating a cranial CT scan that showed improvements (Figure 2), the neurology team indicated that the paresis was a reversible hygroma secondary effect. The patient was discharged the next day (ninth postoperative day) with instructions for abundant hydration, caffeine intake, and analgesia as needed. Seven days later (on the 16th postoperative day), the strabismus and diplopia resolved without any reported complications.

## Discussion

Subdural hygromas consist of collections of clear yellowish or blood-tinged fluid that can develop within the subdural space following trauma to the dura [2,5,7,9]. These are commonly seen following head injury but can also occur as a complication of neurosurgery or neuraxial anesthesia [7,9,10]. The dynamic relationship, in the constant craniospinal compartment, between the volumes of the central nervous system, the blood volume, and the CSF is the basis for intracranial and spinal hygroma formation [6]. According to the Monro-Kellie doctrine, a CSF leak into the epidural space via a dural defect leads to compensatory vasodilatation of the pachymeningeal blood vessels. The formation of intracranial hygroma is believed to result from the effusion of interstitial fluid through these dilated vessels, driven by a pressure gradient [2,3,7,8]. Congestion and tearing of the bridging veins in the subdural space due to traction by down-ward brain displacement is another possible explanation [5] and can be the mechanism by which hygromas can be complicated by hematomas [3,6]. These phenomena are more commonly observed in both infants, whose brains are highly compressible, and the elderly, where brain atrophy creates empty spaces where fluid can accumulate [10]. Intracranial subdural hygromas can be unilateral or bilateral, are more often located in the frontal or temporal regions, and rarely extend caudally [7,10].

There is limited reporting on the formation of subdural hygroma following epidural analgesia in labor. While its true incidence is not well understood and believed to be rare [7], it is possible to be more common than recognized, particularly in asymptomatic patients and after unnoticed dural puncture [7,8]. Most patients with subdural hygromas are asymptomatic or clinically “silent” [10]. However, some individuals may exhibit orthostatic headache, neck rigidity, photophobia, nausea or vomiting, focal neurological deficits, seizures, and even reduced level of consciousness [2,7,10]. The amount of CSF outside the arachnoid space is likely to play a role in the severity of the symptoms [7]. Persisting or returning headaches following initial resolution, loss of their postural nature, or association with hemodynamic changes should prompt neuroradiological investigation to assess sequelae of intracranial hypotension [2,3,7]. Our patient presented with postural headache, as well as photophobia, convergent strabismus, and paresthesia on the right hemiface. Despite having no history of dural puncture, the presence of these neurological symptoms, combined with attempted neuraxial anesthesia, prompted us to conduct an imaging investigation, which ultimately led to the diagnosis of hygromas.

A major concern of subdural hygromas is the progression to a chronic subdural hematoma [2,9], especially if the conditions leading to their formation persist over several weeks [8]. Following the well-recognized subdural hygroma formation secondary to traumatic brain injury, progression to subdural hematoma is seen in 8.2% of patients [2,7]. In other studies, up to 47% of subdural hygromas are complicated with subdural hematomas, which carry a reported mortality rate between 17% and 29% [3]. In severe untreated cases, significant intracranial hypotension with ongoing CSF leak may progress to cerebellar herniation [7]. Differentiating subdural hygroma from a chronic subdural hematoma on CT scans is a subject of debate, and MRI is generally considered to be the preferred imaging modality [2,7]. Due to the unavailability of an MRI machine at our hospital and the urgent need for diagnosis, we chose the CT scan as the preferred imaging study.

A conservative approach, including bed rest, caffeine, hydration, and analgesia, can safely be used to manage uncomplicated subdural fluid collections (hematomas or hygromas) associated with CSF hypotension [3,7]. The effused fluid will reabsorb and be redistributed [7], although it may take up to three months to resolve [3]. If the patient's symptoms persist despite these measures, an epidural blood patch may be necessary [3,7]. This technique is thought to address intrathecal hypotension by either repairing the tear in the dura mater or displacing an equivalent volume of CSF [7]. Surgical treatment, with craniotomy or burr hole evacuation, should be considered in persisting or complicated cases, although it is seldom required, even for large subdural fluid collections exerting significant mass effect [3,7,10]. The advent of new or worsening symptoms demands for additional neurological imaging to determine whether there is a progression in intracranial pathology [7]. We attribute the favorable resolution of this case to the early recognition and prompt treatment with an epidural blood patch, after obtaining informed consent, which was made possible due to our high level of suspicion. The patient was discharged successfully, which was crucial in enabling her to care for her newborn baby and she experienced complete resolution of her symptoms by the 16th postoperative day.

## Conclusions

Although reportedly rare, subdural hygromas can occur as a complication of neuraxial anesthesia during labor. This case highlights the importance of structured follow-up care, even after unsuccessful attempts at neuraxial anesthesia, which can translate into an unrecognized dural puncture. Anesthesiologists should be mindful of the possibility of subdural hygromas and hematomas in parturients who present with persistent headaches and neurological or hemodynamic disturbances. Successful management can be achieved conservatively, with bed rest, hydration, caffeine, and analgesia, or, in more severe refractory cases, with an epidural blood patch. Maintaining a high index of suspicion for pathological sequelae, particularly in these cases, and having a low threshold for imaging and investigation can help prevent unfavorable or even fatal outcomes.
